# Service- and practitioner-level variation in non-consensual dropout from child mental health services

**DOI:** 10.1007/s00787-019-01405-6

**Published:** 2019-09-21

**Authors:** Julian Edbrooke-Childs, Jan R. Boehnke, Victoria Zamperoni, Ana Calderon, Andy Whale

**Affiliations:** 1 Evidence Based Practice Unit, UCL and Anna Freud National Centre for Children and Families, 12 Maresfield Gardens, London, NW3 5SU UK; 2 School of Nursing and Health Sciences, University of Dundee, London, DD1 4HJ UK; 3 Child Outcomes Research Consortium, Anna Freud National Centre for Children and Families, London, UK

**Keywords:** Adolescent mental health, Risk adjustment, Service-level variation, Non-consensual dropout

## Abstract

Non-attendance of mental health service appointments is an international problem. In the UK, for example, the estimated cost of non-attendance in child mental health services is over £45 million (US dollar 60.94 million) per annum. The objective of this study was to examine whether there were service- and practitioner-level variation in non-consensual dropout in child mental health services. This was an analysis of routinely collected data. Service-level variation (as services covered different geographic areas) and practitioner-level variation were examined in *N* = 3622 children (mean age 12.70 years; SD 3.62, 57% female, 50% white or white British) seen by 896 practitioners across 39 services. Overall, 35% of the variation in non-consensual dropout was explained at the service level and 15% at the practitioner level. Children were almost four times more likely to drop out depending on which service they attended (median odds ratio = 3.92) and were two-and-a-half times more likely to drop out depending on which practitioner they saw (median odds ratio = 2.53). These levels of variation were not explained by levels of deprivation in areas covered by services or by children’s demographic and case characteristics. The findings of the present research may suggest that, beyond service-level variation, there is also practitioner-level variation in non-consensual dropout in child mental health services.

**Electronic supplementary material:**

The online version of this article (10.1007/s00787-019-01405-6) contains supplementary material, which is available to authorized users.

## Introduction

Non-attendance of mental health service appointments is an international problem [13]. In the United Kingdom (UK), for example, the estimated cost of non-attendance in child mental health services is over £45 million (US dollar 60.94 million) per annum with an estimated 157,000 missed appointments in 2016 [1]. To effectively target and prevent non-attendance, it is critical to understand the sources of variation in non-attendance.

In the context of child mental health services, appointment non-attendance is more complex than the conceptualisation in adult health services, because it may result from carers not bringing children to appointments [18], potentially compromising the child’s wellbeing with corresponding implications for safeguarding [3]. Health service guidance on appointment non-attendance in child services focusses on parental factors in general, with a lack of definitions of non-attendance or recommendations for specific sanctions [4].

There have been recent calls for child mental health services in the UK to be structured according to a young person’s needs, but they are currently structured according to four tiers: Tier 1—non-specialist support for common problems of childhood (e.g. sleeping), Tier 2—specialist support provided in primary care settings (e.g. bereavement), Tier 3—specialist multidisciplinary child and adolescent mental health teams based in local clinics dealing with more complex problems (e.g. autism), and Tier 4—specialised day and inpatient units for patients with more severe mental health problems [26]. The current policy priority is to improve the provision of child mental health and wellbeing support services [9], which has been described as a postcode lottery, meaning that there is large geographic variation in health-care quality [5]. It is vital to understand variation in health-care quality, such as in non-consensual dropout (i.e. when discharged against professional advice).

Given the prevalence and cost of non-consensual dropout highlighted above, research on non-consensual dropout is urgently needed. Evidence from previous reviews and meta-analyses suggest that certain factors are associated with increased likelihood of non-consensual dropout, such as disagreement on presenting problems, goals, or approaches; weaker therapeutic alliance; or lack of improvement [6, 19, 21]. However, previous systematic reviews have not examined practitioner-level variation: differences in dropout between practitioners and the likelihood of dropping out depending on which practitioner a young person saw. The systematic review conducted for the present research (see supplementary material) identified studies reporting 4–15% practitioner-level variation in non-consensual dropout rates in adult settings [10, 20, 25, 29]. Only one study was conducted in child mental health settings, which found ≤ 2% practitioner-level variation in non-consensual dropout rates across 406 adolescents (seen by 144 practitioners) taking part in a randomised control trial of treatments for depression [17].

The aim of the present research was to examine practitioner- and service-level variation in non-consensual dropout in child mental health services.

## Methods

### Participants and procedure

Data were collected from services that were part of a national transformation initiative in England, which embedded best practice by training practitioners in key evidence-based interventions and encouraging routine use of feedback and outcome monitoring (Children and Young People’s Improving Access to Psychological Therapies or CYP IAPT). Data were submitted by 75 geographic partnerships or services situated across England, each made up of government-funded and voluntary sector providers [27]. Services made quarterly submissions of patient-level data, and data submissions occurred between January 2012 and October 2015.

Cases were deemed eligible for analysis if they: (1) were recorded as “closed”, (2) had a recorded reason for case closure, (3) had at least one recorded event with a practitioner identifier, (4) had at least one item on the measure of presenting problems completed within 56 days of the recorded period of contact start, and (5) had data on more than one child per service. This resulted in a total sample of *N* = 3622 children^1^ with an average age of 12.70 years (SD 3.62), 57% female, 50.08% white or white British. This included data from 896 practitioners (with 1–80 children per practitioner) and 39 services (range 2–584 children and 1–123 practitioners per service).

As measures were taken from a secondary analysis of anonymised routinely collected data, ethical review was not relevant [16].

### Measures

#### Case characteristics

Age, gender, and ethnicity were recorded by services as part of routine data recording. Gender was coded female (1) vs. male (0). Ethnicity was coded as white or white British (1) vs. any other ethnicity (0), including missing or not stated (*n* = 667). Presenting problems were identified using an algorithm [28] based on 30 items of the clinician-rated Current View (CV) questionnaire [11]. The algorithm categorises children into 18 mutually exclusive needs-based groups. However, we used ten groups because no child was identified in the “Neurodevelopmental Assessment” group and eight groups were collapsed into a “low-frequency” group as they occurred ≤ 1% (i.e. “Bipolar Disorder”, “Eating Disorders”, “Obsessive Compulsive Disorder”, “Psychosis”, “Autism”, “Co-occurring Behavioural and Emotional Difficulties”, “Post Traumatic Stress Disorder”, and “Social Anxiety Disorder”).

#### Deprivation

In line with approaches used in previous studies [7, 24], we matched data on services to the normalised Income Deprivation Affecting Children Index (IDACI) to generate an average rank based on the rank of all Lower Layer Super Output Areas in each service’s catchment area [8]. The IDACI is based on the geographical area covered by a service and is an area-level indicator of deprivation widely used in policy research [23].

### Measures

#### Non-consensual dropout

Case closure reasons were recorded by practitioners. These were recoded into non-consensual dropout (1; discharged against professional advice, patient non-attendance) vs. consensual dropout (0; discharged on professional advice, transferred to other health-care provider medium secure unit, transferred to other health-care provider high secure unit, transferred to other health-care provider not medium/ high secure, transferred to adult mental health service, patient moved out of area, patient died).

### Analytic strategy

To investigate the amount of practitioner-level variation in non-consensual dropout, multilevel logistic modelling (MLM) was performed in STATA 12 [22]. Four models were estimated: (1) to examine service-level variation, a null model examining service-level variation in non-consensual dropout as services cover different geographic areas; (2) to examine practitioner-level variation, a null model examining service- and practitioner-level variation in non-consensual dropout; (3) to examine whether service-level deprivation explained service-level variation in dropout, grand mean centred IDACI rank was added;^2^ and (4) case characteristics (grand mean centred age, female, white or white British, and dummy coded presenting problems^3^ where the “Advice” group, referring to young people for whom clinicians rated a maximum of one problem as moderate, was selected as the reference category, as it was the largest group; i.e. *n* = 1755 or 48.45% as shown in Table 1) were added to examine whether case characteristics accounted for service- or practitioner-level variation in non-consensual dropout. Intraclass correlations and median odds ratios were calculated to quantify the clustering effects due to practitioners and services [12, 14].

**Table 1:** Multilevel logistic regressions with service level, practitioner level, and case characteristics explaining variation in non-consensual dropout

Parameter estimates	Model 1		Model 2		Model 3		Model 4	
	Estimate (SE)	OR	Estimate (SE)	OR	Estimate (SE)	OR	Estimate (SE)	OR
Fixed effects								
Intercept	− 2.32 (0.26)***	0.10	− 2.65 (0.29)***	0.07	− 2.61 (0.28)***	0.07	− 2.98 (0.33)***	0.05
IDACI					0.00 (0.00)	1.00	0.00 (0.00)*	1.00
Female vs. male							− 0.00 (0.13)	1.00
Age							0.06 (0.02)**	1.06
White or white British vs. other							0.15 (0.13)	1.16
ADHD (2.73%) vs. advice							0.50 (0.39)	1.65
Behavioral and/or conduct disorders (3.29%) vs. advice							0.44 (0.34)	1.55
Depression (4.00%) vs. advice							0.68 (0.34)*	1.98
Difficulties not covered by other groupings (10.80%) vs. advice							0.07 (0.23)	1.07
Difficulties of severe impact (6.35%) vs. advice							0.34 (0.27)	1.40
Co-occurring emotional difficulties (6.76%) vs. advice							0.27 (0.26)	1.32
Generalized anxiety disorder and/or panic disorder (3.40%) vs. advice							0.03 (0.38)	1.03
Self-harm (5.25%) vs. advice							0.85 (0.31)**	2.33
Low frequency (8.97%) vs. advice							0.64 (0.24)**	1.89
Variance components								
Practitioner level, *SD*			0.97 (0.12)		0.97 (0.12)		0.98 (0.12)	
Service level, *SD*	1.33 (0.22)		1.43 (0.25)		1.35 (0.23)		1.42 (0.25)	
Quantification of cluster effects								
Practitioner level, *ICC, MOR*			0.33, 3.92		0.30, 3.63		0.32, 3.86	
Service level, *ICC, MOR*	0.35, 3.56		0.15, 2.53		0.16, 2.53		0.15, 2.55	

*N* = 3622. Advice *n *=1755 (48.45%), percentages of groups in parentheses

*IDACI* Income Deprivation Affecting Children Index average rank, *ICC* intraclass correlation coefficient, *MOR* median odds ratio

* = *p *< 0.05. ** = *p *< 0.01. *** = *p *< 0.001

## Results

According to the intraclass correlation coefficient, in Model 1, 35% of the variation in non-consensual dropout was explained at the service level, which remained mostly unchanged across subsequent models. In Model 2, 15% of the variation in non-consensual dropout was explained at the practitioner level, in addition to that explained at the service level. Here, children were almost four times more likely to drop out depending on which service they attended (median odds ratio = 3.92), and children were two-and-a-half times more likely to drop out depending on which practitioner they saw (median odds ratio = 2.53). In Model 3, adding service-level IDACI did not significantly improve the model fit: likelihood ratio test: *χ*^2^(1) = 1.53, *p* > 0.05. In Model 4, adding patient-level case characteristics did not significantly improve the model fit: likelihood ratio test: *χ*^2^(12) = 14.09, *p* > 0.05.

## Discussion

The aim of the present research was to examine service-level and practitioner-level variation in non-consensual dropout in child mental health services. Overall, about half of the variance was due to differences between services and practitioners, with median odds ratios indicating that, depending on the service, patients could be nearly four times more likely to drop out and, depending on the practitioner, about two-and-a-half times more likely to drop out.

The findings of the present research are consistent with the studies identified in the systematic review for the present research, which reported 4–15% practitioner-level variation in non-consensual dropout rates in adult settings [10, 20, 25, 29]. We found much larger practitioner-level variation than the one previous study in a child mental health setting, which found ≤ 2% practitioner-level variation in 406 young people seen by 144 practitioners [17]. However, differences in the patterns of finding could be due to differences in sample sizes of young people and practitioners, characteristics of the samples (e.g. the previous study examined depression only), or study design, as the previous study examined data from a randomised control trial and the present study was an analysis of routine data. Surprisingly, case characteristics were not significant predictors of dropout in the present study. We also investigated whether deprivation (as estimated by the service’s geographical location) had an impact on non-consensual dropout, which was identified as a relevant predictor of between-service variation in adult outcomes of psychological therapies in the UK [7]. Although service-level deprivation does not seem to be a relevant variable to explain differences in non-consensual dropout as adding it did not improve the overall model, deprivation could still prove to be a relevant predictor of outcome. Future studies should examine other indicators of outcome (such as improvement in mental health symptoms) and other indicators of deprivation (such as family income), which were not available in the present data.

Evidence from previous reviews and meta-analyses suggest that certain factors are associated with increased likelihood of non-consensual dropout [6, 19, 21]. Factors include those related to pre-treatment child characteristics—such as ethnic minority status and young people with externalising problems—and treatment and therapist factors—such as more cancellations or missed appointments, lower perceived relevance of treatment, lower therapeutic relationship, and similarly lower levels of therapist compassion. The implications of the present research suggest that providers should focus on service- and practitioner-level barriers. In particular, a recent scoping review on service-level barriers to access and engagement with youth mental health services [2] emphasised the provision of available and flexible services that respond to the needs of families (e.g. to minimise the need for parents/carers to take time off work to attend appointments), as was information about services, short waiting times, simple administrative processes, and addressing users’ expectations of providers’ attitudes (e.g. concerns that practitioners and services will not be compassionate or respectful and that parents/carers will need to convince the provider that their child has a problem in the first instance). One recommendation of the findings of the present research may be that services and practitioners should review the information provided to service users about their interventions, highlighting the approaches they use to treat children and families with compassion. Practitioners in particular could review their approaches for seeking feedback from families about how they are experiencing therapy to identify families at an early stage that might be more likely to non-consensually dropout.

Limitations should be considered when interpreting the findings of the present research, particularly regarding the use of routinely collected data [27]. Without a randomised allocation of patients to services and practitioners, inferences of causation should not be made. Incomplete data recording meant that we were unable to examine other characteristics that may be relevant to non-consensual dropout, such as therapy and practitioner type. The use of routinely collected datasets means that there may be some variation in how data were collected and recorded, as individual services may have collected and coded information differently, which has been noted as a limitation in physical health settings [15]. Differences in data recording could have resulted in some services being more likely to not record practitioner identifiers and some practitioners being more likely to not record closed cases due to non-consensual dropout. Although the present study was based on a large national dataset, findings may not generalise to other services in the UK. In particular, as data were drawn from a particular service initiative, there may be selection bias, in that services involved with the initiative may have different dropout rates to services not involved with the initiative. Moreover, although services covered different geographic regions, we did not have specific data on postcodes, so deprivation indicators for service areas may not have fully captured the level of deprivation of all service users. Additionally, the IDACI average rank means that polarised areas tend to “average out” so it may not capture areas of high deprivation, although the pattern of results was the same when other indicators of IDACI and the index of multiple deprivation were used. Future studies should replicate the findings of the present research using larger samples.

Nevertheless, we controlled for a number of demographic and case characteristics and, regardless of reasons for the practitioner and service variation in dropout rates, the absolute sizes of both effects merit further investigation. The findings of the present research are an important contribution to the literature, as only one other study has examined practitioner-level variation in non-consensual dropout from child mental health services.

The findings of the present research suggest that clear guidelines on non-consensual dropout and greater reporting and transparency of non-consensual dropout rates are needed. The findings of the present research may be useful for the early identification of higher than expected dropout rates at the service and practitioner level, to enable access to appropriate support. Children were almost four times more likely to drop out depending on which service they attended and were two-and-a-half times more likely to drop out depending on which practitioner they saw in the present study. These levels of variation were not explained by levels of deprivation in areas covered by services or by young people’s demographic characteristics and presenting problems. Understanding variation in non-consensual dropout is vital given current policy on improving the provision of child mental health and wellbeing support services.

## Electronic supplementary material

Below is the link to the electronic supplementary material.

Supplementary material 1 (PDF 204 kb)

## Footnotes

1 The present research used a national dataset collected from services involved in a best practice transformation programme described elsewhere [27]. In this dataset, pseudonymised data are uploaded according to the episodes of care. Therefore, it is possible that a child may have been included under more than one episode of care.

2 The pattern of results was the same when other indicators of deprivation were used (i.e. IDACI average score, index of multiple deprivation (IMD) average score, IMD average rank, and IMD extent score).

3 The pattern of results was the same when individual presenting problems were used.
