# Supplementary material for: Service- and practitioner-level variation in non-consensual dropout from child mental health services
Source: Eur Child Adolesc Psychiatry. 2019 Sep 21;29(7):929–34. doi: 10.1007/s00787-019-01405-6 (PMC7321904; doi:10.1007/s00787-019-01405-6)
Supplement: Supplementary file 1 — Supplementary material 1 (PDF 204 kb) [file 787_2019_1405_MOESM1_ESM.pdf]

Service- and practitioner-level variation in non-consensual dropout from child mental health services

Supplementary material
Systematic review

PRISMA 2009 Flow Diagram

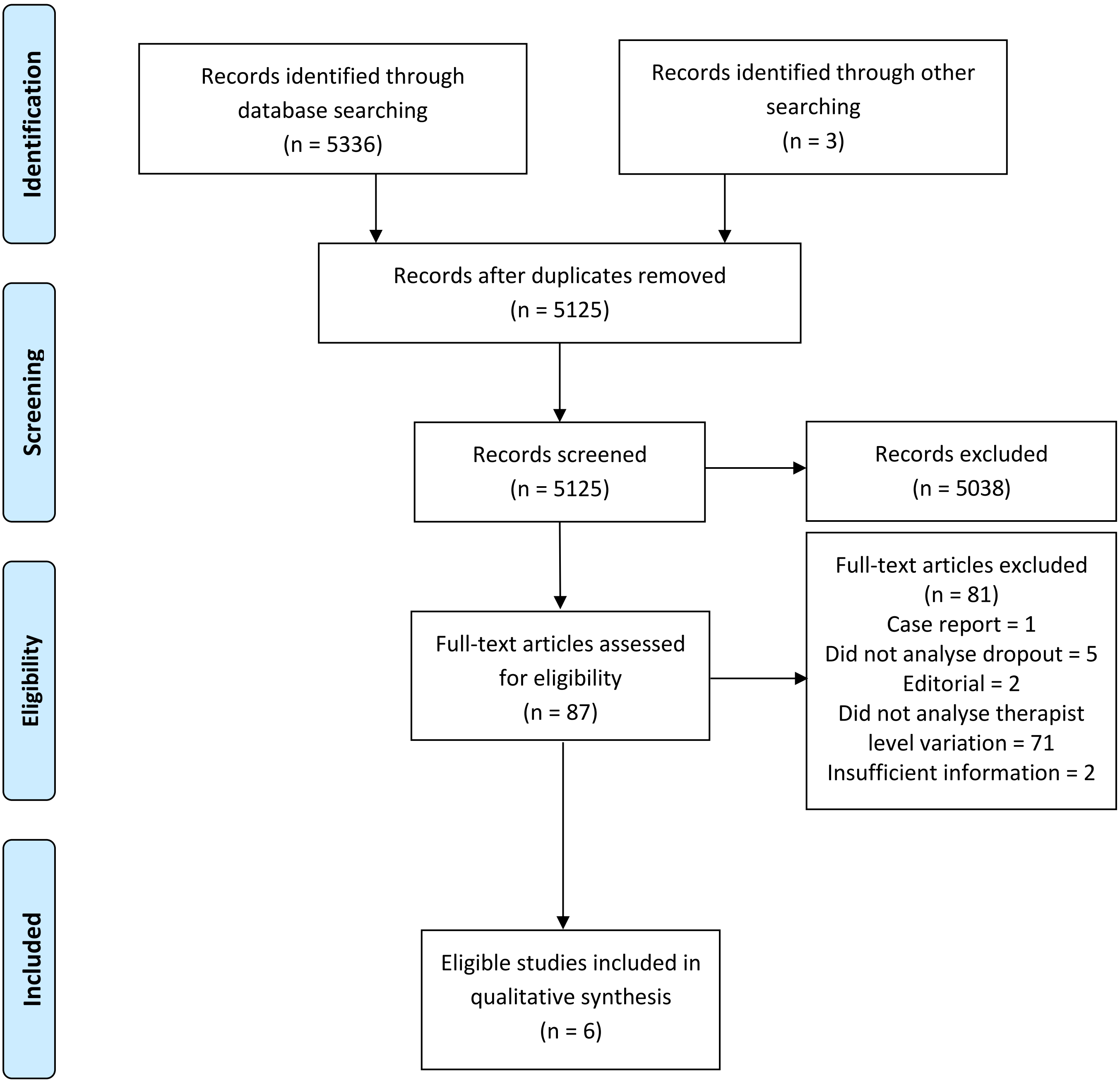

PICOS statement

Participants	Intervention	Comparator	Outcome	Study design
Mental health, no restriction on age	Therapist effects	Patient-level variation, service-level variation	Non-consensual dropout	Cohort, observational, controlled trial

Screening

TI/AB: exclude obviously irrelevant hits

1. Not English
2. Not published
3. Not empirical
4. No mention of mental health OR secondary mental health to physical health condition
5. No mention of non-consensual dropout OR only reporting dropout rates
6. Include if too little information to decide

Citation tracking

Hand search of included journals (2006-2016)

- Behaviour research and therapy
- Psychotherapy research
- Clinical psychology and psychotherapy

Reference search of included papers

- 1 paper identified from citation tracking

A second author (AC) screened 10% of titles/abstracts and 10% of full-texts.

Databases, search terms, and search results

Search terms derived from Zimmerman et al. (2016)*, O'Brien et al. (2009), Swift and Greenbergh (2010), and Huppert et al., (2014)*.

Baseline terms

Participants	Intervention	Comparator	Outcome	Study design
Mental health	Therapist effects, mixed effect logistic regression, therapist variables	Patient-level variation, service-level variation	Non-consensual dropout, engagement, disengagement, attrition, continuance, termination	Cohort, observational, controlled trial

PsycINFO

09.06.16: 451

EN

.sh=subject heading; .mp=title, abstract, heading word, table of contents, key concepts, original title, tests & measures; .md=methodology; .id=key concepts (IV, DV, population)

Participants	Intervention	Comparator	Outcome	Study design
(exp mental health/ OR exp community	(exp therapist characteristics/).sh OR (therapist		(exp dropouts OR treatment compliance/	(quantitative study OR empirical study

mental health service/ OR exp community psychiatry/ OR exp emotional adjustment/ OR exp anxiety disorder/ OR exp agoraphobia/ OR exp mental health programs/ OR exp mental health services/ OR exp primary mental health prevention/ OR exp well being/ OR exp abnormal psychology/ OR exp attention deficit disorder/ OR exp attention deficit disorder with hyperactivity/ OR exp behavior disorders/ OR exp communication disorders/ OR exp conduct disorder/ OR exp emotional adjustment/ OR exp emotional disturbances/ OR exp intellectual development disorder/ OR exp learning disorder/ OR exp psychiatric patients/ OR exp psychopathology/ OR exp suicide/ OR exp thought disturbances/ OR exp psychotherapeutic outcomes/ OR exp mental health	effect* OR therapist variable OR practitioner effect* OR practitioner variable OR clinician effect* OR clinician variable OR mixed effect* OR multilevel OR ICC OR intraclass correlation coefficient).mp		OR exp treatment refusal/ OR exp treatment termination/) .sh OR (dropout OR engagement OR disengagement OR attrition OR continuance OR termination OR dna) OR (did not attend) .mp	OR followup study OR longitudinal study OR prospective study OR retrospective study OR field study OR treatment outcome/clinical trial) .md

program evaluation/) .sh				

MEDLINE
10.06.16; 618
EN
Kf=keyword heading word

Participants	Intervention	Comparator	Outcome	Study design
(exp mental health/ OR exp mental disorders/ OR exp mental health services/ OR exp emotional adjustment/ OR exp depression/ OR exp obsessive behavior/ OR exp schizophrenic language/ OR exp self-injurious behavior/ OR exp stress, psychological/ OR exp psychopathology/ OR exp treatment outcome/) .mesh	(exp multilevel analysis/).mesh OR (therapist characteristics OR practitioner characteristics OR clinician characteristics OR therapist effect* OR therapist variable OR practitioner effect* OR practitioner variable OR clinician effect* OR clinician variable OR mixed effect* OR multilevel OR ICC OR intraclass correlation coefficient) .kf,ti,ab		(exp patient dropouts/ OR exp patient compliance/ OR exp treatment refusal/) .mesh OR (dropout OR engagement OR disengagement OR attrition OR continuance OR termination OR dna) .kf, ti, ab OR (did not attend). kf, ti, ab	(exp case-control studies/ OR exp cohort studies/ OR exp controlled before-after studies/ OR exp historically controlled study/ OR exp cross-sectional studies/ OR exp interrupted time series analysis/ OR exp multicentre studies as topic/ OR exp longitudinal studies/) .mesh

EMBASE
10.06.16; 2,888
EN

Participants	Intervention	Comparator	Outcome	Study design
(exp mental health/ OR exp mental health service/ OR exp social psychiatry/ OR exp mental disease/ OR exp mental health care/ OR exp	(exp health care personnel/ OR exp multilevel analysis/ OR exp correlation coefficient/).sh OR (therapist effect* OR therapist variable OR		(exp patient dropouts/ OR exp patient compliance/ OR exp treatment refusal/) .sh OR (dropout OR engagement OR disengagement OR attrition OR	(exp quantitative study/ OR exp empiricism/ OR exp follow up/ OR exp clinical study/ OR exp field study/) .sh

well being/ OR exp mental patient/ OR exp suicidal behavior / OR exp treatment outcome/ OR exp program evaluation/) .sh	practitioner effect* OR practitioner variable OR clinician effect* OR clinician variable OR mixed effect* OR multilevel OR ICC OR intraclass correlation coefficient). kw		continuance OR termination OR dna) .kw OR (did not attend) .kw	

Cochrane
13.06.16; 663

Participants	Intervention	Comparator	Outcome	Study design
(mental health OR mental disorders OR mental health services OR emotional adjustment OR depression OR obsessive behavior OR schizophrenic language OR self-injurious behavior OR stress, psychological OR psychopathology OR treatment outcome) .mesh	(multilevel analysis).mesh OR (therapist characteristics OR practitioner characteristics OR clinician characteristics OR therapist effect* OR therapist variable OR practitioner effect* OR practitioner variable OR clinician effect* OR clinician variable OR mixed effect* OR multilevel OR ICC OR intraclass correlation coefficient) .kw, ti, ab		(patient dropouts OR patient compliance OR treatment refusal) .mesh OR (dropout OR engagement OR disengagement OR attrition OR continuance OR termination OR dna) .kw, ti, ab OR (did not attend). Kw, ti, ab	(case-control studies OR cohort studies OR controlled before-after studies OR historically controlled study OR cross-sectional studies OR interrupted time series analysis OR multicentre studies as topic OR longitudinal studies) .mesh

Web of Science (topic [TS] = title, abstract, author keywords, keywords plus)
08.06.16: 1,379
EN

Participants	Intervention	Comparator	Outcome	Study design
TS=(mental	TS=(therapist		TS=(dropout	TS=(cohort OR

health OR mental disorder OR mental illness OR psychotherapy research OR depress* OR anxiety OR mood disorder OR bipolar disorder OR conduct problem OR conduct disorder OR hyperactive* OR internalising OR internalizing OR externalising OR externalizing OR self harm)	effect OR therapist variable OR practitioner effect OR practitioner variable OR clinician effect OR clinician variable OR mixed effect OR multilevel OR ICC OR intraclass correlation coefficient)		OR engagement OR disengagement OR attrition OR continuance OR termination OR did not attend OR dna)	observational OR control trial OR before and after)

Data extraction

Citation	Participants	Intervention	Comparator	Outcomes	Study design
(Huppert et al., 2014)	205 patients with primary panic disorder with or without agoraphobia randomized to CBT, of whom 183 had sufficient data (70 men, 113 women); 14 doctoral-level therapists with data on 4 or more patients (7 men, 7 women; 13 psychologists, 1 psychiatrist)	CBT arms of a RCT (CBT only and CBT with imipramine/placebo)	N/A	Variance in completion status was NS (S^2=0.16, p=0.07); variance 4.3% of variance in number of completed sessions explained at therapist level (S^2=0.39, p=0.19)	Secondary analysis of RCT data
(Zimmermann, Rubel, Page, & Lutz, 2016)	Patients with dropout or completion recorded 2007-2014=766, with Brief Symptom Inventory=742, >=3 session=737, >=3 patients per therapist=707 (63.4% women, mean age=35.93, SD age=12.7,	Weekly treatment informed by a therapy feedback system	N/A	Therapist effect ICC=5.72%; therapist effect differed significantly from zero (chi-squared (1,707)=6.87, p<.001)	Cohort, routinely collected data from an outpatient clinic in Germany

	97% with at least one diagnosis, of which the most frequent was major depressive disorder at 42.3%); 66 therapists with an average of 10.71 patients each (SD=6.32), trainees, 84.8% female, mean age = 29.44 years, SD age=5.65				
(Werbart, Andersson, & Sandell, 2014)	1498 cases, of whom 204 were nonstarters, 535 were data collection drop outs, and 5 clinics contributed less than 15 cases; 750 cases in 8 clinics of whom 495 remained in treatment; patient-initiated drop out = 148, therapist-initiated dropout = 107, continuation =	Psychotherapy type indicated for 252 cases, with the most common being PDT (149), INT (48) and CBT (43) with the remaining being other (12)	N/A	ICC = .13 for patient-initiated drop out vs continuation and .39 for therapist-initiated dropout	Naturalistic setting of routine mental healthcare with data collected between January 2007 and February 2010 collected as part of a Quality Assurance of Psychotherapy in Sweden programme

	495; 487 had a psychiatric diagnosis: axis I for 464 patients, axis II for 141 patients and comorbid axis I and II for 99				
(Saxon, Barkham, Foster, & Parry, 2016)	70,245 clients by 1,059 therapists in 35 UK counselling and clinical psychology services between 1999 and 2008, of whom therapists were selected if they provided treatment ending information >90% patients which resulted in a final sample of 10,521 patients by 85 therapists from 14 sites; patients = 71.2% female, mean age = 40.3 years (SD = 13 years), 4.6% non white, 76.8% had	Therapy type not reported	N/A	Therapists recorded completion or dropout at case closure, 33.8% drop out; PrI 12.6% (9.1-17.4)	Naturalistic study using routinely collected data

	some level of depression and 82.7% had some level of anxiety				
(Xiao et al., 2017)	10,147 clients by 481 therapists in US college counselling centre from 2010-2012; patients had at attended at least 1 session with complete pre-post measures within 30 days; patients = 22.62 years (SD=5.01), 66.7% female, 73.3% White/Caucasian; 15.9% dropout (n=1617)	Therapy type not reported	N/A	Nonattendance of the last scheduled session for an individual's course of therapy as recorded by each center's electronic medical records system, and failure to achieve at least an RCI change on pre-post overall measure of distress; 9.11% therapist level variation	Naturalistic study using routinely collected data
(O'Keeffe et al., 2017)	406 patients (74% female, 81% White British, mean (SD) age = 15.59 (1.43)) 144 therapists who saw 1-15 patients across 15 services 37% dropout (p.9)	Brief Psychosocial Intervention $n_{therapists}$ = 62, CBT $n_{therapists}$ = 44, Short-Term Psychoanalytic Psychotherapy $n_{therapists}$ = 38	N/A	Ended without the mutual agreement of the therapist reported by therapists at the end of treatment; ≤ 2%	Secondary analysis of RCT data

Risk of bias assessment (no. of stars, description and page no.)

**Ref.**	**Representativeness**	**Selection**	**Ascertainment**	**Absence at start**	**Comparability of cohorts**	**Assessment of outcome**	**Length of follow-up**	**Adequacy of follow-up**
(Huppert, et al., 2014)	* 92% of patients who were enrolled in the study and entered treatment were included in the analysis (p.28)	* non exposed cohort drawn from the same community as the exposed cohort (p.28)	*1 therapist and investigator reasons for drop out recorded (White et al., 2010) (p.668)	* demonstration that outcome of interest was not present at start of study as all patients had entered treatment (p.28)	* patients received the same treatment and adherence was monitored (p.29)	See ascertainment	* data collected from patients at the end of the first phase of the trial (p.28)	* 0% dropout from data collection
(Zimmermann, et al., 2016)	* 92% of patients with drop out or completion between 2007-14 were in the included sample (p.3)	* non exposed cohort drawn from the same community as the exposed cohort (p.3)	0: ascertainment of exposure by self report (therapist evaluation) (p.4)	* demonstration that outcome of interest was not present at start of study as patients had to have attended at least 3 sessions (p.3)	0: no discussion of types of treatment received (p.4)	See ascertainment	* drop out assessed at the end of treatment (p.4)	* 0% dropout from data collection
(Werbart, et al., 2014)	0: selected group of users: included sample of 64% of total sample, no	* non exposed cohort drawn	0: ascertainment of exposure by written self	* demonstration that outcome of interest	* no differences in drop out vs. continuation in	See ascertainment	* drop out assessed at post-	0: 36% dropout from data collection

	comparison of baseline characteristics of included and excluded samples (p.727)	from the same community as the exposed cohort (p.727)	report (therapist questionnaire) (p.726-7).	was not present at start of study as those who did not begin treatment were excluded (p.727)	types of therapy received (p.730)		treatment (p.726)	
(Saxon, et al., 2016)	* included therapists with completion data for >90% of patients and participant selection clearly outlined (p.3-4)	* non exposed cohort drawn from the same community as the exposed cohort (p.3-4)	0: ascertainment of exposure by self report (therapist evaluation) (p.4)	* demonstration that outcome of interest was not present at start of study as those who did not begin treatment were excluded (p.3-4)	0: no discussion of types of treatment received (p.3)	See ascertainment	* drop out assessed at post-treatment (p.4)	* 0% dropout from data collection (p.4)
(Xiao, et al., 2017)	0: selected group of users: included sample of 8.34% of total sample, no comparison of baseline	* non exposed cohort drawn from the same community as the exposed cohort	* ascertainment of exposure clinic dataset and reliable change on self-report measure (p.426)	* demonstration that outcome of interest was not present at start of study as those who did not begin treatment	0: no discussion of types of treatment received (p.426-7)	See ascertainment	* drop out assessed at post-treatment (p.426)	? unclear how many patients had attended more than 1 session but did not complete measures

		(p.426)		were excluded (p.426)				(p.426)
(O’Keeffe, et al., 2017)	* selected group of users: included sample of 97% of total sample (p.4)	* non exposed cohort drawn from the same community as the exposed cohort (p.3-4)	0: ascertainment of exposure by self report (therapist evaluation) (p.4)	* demonstration that outcome of interest was not present at start of study as those who did not begin treatment were excluded (p.4)	* analyses accounted for therapy received (p.6)	See ascertainment	* drop out assessed at post-treatment (p.4)	* 10% dropout from data collection

Note. ? = unclear. 0 = high risk of bias. * = low risk of bias.

References

Huppert, J. D., Kivity, Y., Barlow, D. H., Gorman, J. M., Shear, M. K., & Woods, S. W. (2014). Therapist effects and the outcome-alliance correlation in cognitive behavioral therapy for panic disorder with agoraphobia. *Behaviour Research and Therapy, 52,* 26-34.

O'Keeffe, S., Martin, P., Goodyer, I. M., Wilkinson, P., Consortium, I., & Midgley, N. (2017). Predicting dropout in adolescents receiving therapy for depression. *Psychotherapy Research,* 1-14. doi: 10.1080/10503307.2017.1393576

Saxon, D., Barkham, M., Foster, A., & Parry, G. (2016). The Contribution of Therapist Effects to Patient Dropout and Deterioration in the Psychological Therapies. *Clinical Psychology & Psychotherapy*. doi: 10.1002/cpp.2028

Werbart, A., Andersson, H., & Sandell, R. (2014). Dropout revisited: Patient-and therapist-initiated discontinuation of psychotherapy as a function of organizational instability. *Psychotherapy Research, 24*(6), 724-737.

White, K. S., Allen, L. B., Barlow, D. H., Gorman, J. M., Shear, M. K., & Woods, S. W. (2010). Attrition in a Multicenter Clinical Trial for Panic Disorder. *The Journal of Nervous and Mental Disease, 198*(9), 665-671. doi: 10.1097/NMD.0b013e3181ef3627

Xiao, H., Castonguay, L. G., Janis, R. A., Youn, S. J., Hayes, J. A., & Locke, B. D. (2017). Therapist effects on dropout from a college counseling center practice research network. *Journal of Counseling Psychology, 64*(4), 424-431. doi: 10.1037/cou0000208

Zimmermann, D., Rubel, J., Page, A. C., & Lutz, W. (2016). Therapist effect on and predictors of non-consensual dropout in psychotherapy. *Clinical Psychology and Psychotherapy*. doi: 10.1002/cpp.2022
